# Effects of Functionalization in Different Conditions and Ball Milling on the Dispersion and Thermal and Electrical Conductivity of MWCNTs in Aqueous Solution

**DOI:** 10.3390/nano11051323

**Published:** 2021-05-18

**Authors:** Baasandulam Tserengombo, Hyomin Jeong, Erdenechimeg Dolgor, Antonio Delgado, Sedong Kim

**Affiliations:** 1 Department of Energy and Mechanical Engineering, Graduate School, Gyeongsang National University, Jinju 53064, Korea; baasandulam0902@gmail.com; 2 Department of Energy and Mechanical Engineering, Institute of Marine Industry, Gyeongsang National University, Cheondaegukchi-Gil 38, Tongyeong 53064, Korea; hmjeong@gnu.ac.kr; 3 Department of Chemical and Biological Engineering, School of Engineering and Applied Science, National University of Mongolia, P.O. Box 46A, Ulaanbaatar 14201, Mongolia; erdenechimeg@seas.num.edu.mn; 4 German Engineering Research and Development Center LSTME Busan Branch, Busan 46742, Korea; antonio.delgado@lstme.org; 5 Institute of Fluid Mechanics, Friedrich–Alexander Universität Erlangen-Nürnberg, 91058 Erlangen, Germany

**Keywords:** MWCNTs, functionalization, alkaline treatment, dispersion, thermal conductivity

## Abstract

In this work, the effects of a functionalization method involving different conditions and milling processes on the dispersion and thermal and electrical conductivity of multiwalled carbon nanotubes were studied. The surfaces of MWCNTs were modified using a mixture of sulfuric and nitric acid as an acid treatment and potassium persulfate and sodium hydroxide as an alkaline treatment to achieve more hydrophilic MWCNTs. The morphological and structural investigations were carried out using transmission electron microscopy and Fourier transform infrared spectroscopy. Furthermore, the dispersion characteristics and thermal and electrical conductivity of the as-prepared water-based nanofluids were measured. As a result, the dispersion characteristics revealed that the best dispersion and stability results were obtained for alkaline-treated MWCNTs using potassium persulfate and sodium hydroxide. The thermophysical study using a thermal conductivity analyzer exhibited that the thermal conductivity of the pristine MWCNT nanofluid (0.1 wt%) was enhanced from 603.5 to 610.4 mW/m·K and the electrical conductivity of the raw MWCNT nanofluid was increased from 16.2 to 125.8 μS/cm at 25 °C after alkaline treatment and milling processes, which were performed using planetary ball milling. Regarding the overall results, the milling process and mild alkaline oxidation process are more environmentally friendly, effective, and convenient for the functionalization of CNTs, without requiring any organic solvents or strong acids.

## 1. Introduction

Carbon nanotubes (CNTs) have been highlighted since their discovery due to their unique structure and remarkable mechanical, thermal, and electrical characteristics, which make them excellent candidates in many fields [1,2,3]. Carbon nanotubes are promising materials for various applications, such as energy [4], biological and biomedical [5], environmental [6], composite material [7], electronic [8], optical [9], solar cell [10], and hydrogen storage [11] applications. However, the development of CNT applications has been impeded by their aggregation, as CNTs tend to agglomerate due to their large aspect ratio and van der Waals forces, which lead to dispersion difficulties in dispersion media [12,13].

Therefore, many studies have been focused on how to improve the dispersibility of CNTs.

To solve dispersion problems, many approaches have been attempted, namely ultrasonication, mechanical milling, chemical functionalization, and addition of various surfactants and polymers; good dispersion in a homogeneous solution would expand the scope of their application and improve their properties. Munkhbayar et al. [14] studied the photovoltaic performance of dye-sensitized solar cells with various MWCNT counter electrodes. It was found that the dispersibility of CNT enhances with increasing photovoltaic efficiency. Garg et al. [15] reported an optimum ultrasonication time of 40 min to increase the thermal conductivity of 1 wt% MWCNT nanofluids. Krause et al. [16] studied ball-milled CNTs regarding their morphology and nanotube length. They found that when increasing the ball milling time significantly, this decreased the agglomerate size, shortened the length, and increased the surface area. Additionally, surfactants and polymers can be used to improve the dispersion of nanoparticles [17,18,19]. In our previous work [20], we compared the effects of the surfactants sodium dodecyl sulfate (SDS), sodium dodecyl benzene sulfonate (SDBS), and dodecyl betaine (DB) in aqueous solution regarding their effects on the dispersion and thermal conductivity of CNTs. We found that the dispersion of the nanofluid increased with increasing surfactant concentration; however, the thermal conductivity of the nanofluid with surfactant was lower than the nanofluid without surfactant. Low thermal conductivity may result from surfactant molecules adsorbing onto the surfaces of CNTs, thereby enlarging the resistance between the CNTs and the base fluid.

Nanotube functionalization is the most effective method to improve the dispersion of carbon materials. The functionalization of CNTs using chemical oxidation methods enhances the solubility by incorporating hydrophilic moieties onto the nanotubes. The functional groups are attached to the nanotube ends and side walls via covalent bond formation, thus resulting in more hydrophilic CNTs [21,22]. Oxidizing agents such as nitric acid, sulfuric acid, a mixture of sulfuric acid and nitric acid, potassium permanganate, sulfuric acid in the presence of potassium permanganate, hydrogen peroxide in the presence of nitric acid, hydrogen peroxide, and ozone [23,24,25,26,27,28] are used. Strong acids are the most used oxidants for the chemical functionalization of CNTs [29,30,31]. Acid treatment is effective for CNT functionalization, however it is environmentally unfriendly and leads to equipment corrosion. Recently, reports have emerged outlining a mild oxidation process under alkaline conditions, which is eco-friendlier and has no need for the use of organic solvents and strong acids [32,33].

To the best of the authors’ knowledge, there is only one limited study comparing alkaline and acid treatment methods regarding their effects on the dispersion of MWCNTs. Therefore, in this experimental study, we compared alkaline treatment to conventional acid treatment and considered the effects of milling in terms of the dispersion characteristics, suspension stability, and thermal and electrical conductivity of MWCNT nanofluids.

## 2. Experimental Details

### 2.1. Materials

The Raw MWCNTs measuring ~20 nm in diameter and ~5 μm in length, with greater than 95% purity and less than 3% impurities (Carbon Nanomaterial Technology Co., Ltd., Korea), were used in this experimental study.

Nitric acid (HNO_3_) at a concentration of 63%, sulfuric acid (H_2_SO_4_) at a concentration of 98% (Junsei Chemicals Co., Ltd., Japan), potassium persulfate (K_2_S_2_O_8_) at greater than 99.0% purity (Sigma-Aldrich Co., Ltd., Germany), and sodium hydroxide (NaOH) at greater than 97.0% purity (Junsei Chemicals Co., Ltd., Japan) were used for treatment processes. Distilled water (DW) was used as a base fluid to make nanofluids.

### 2.2. Instruments and Characterization

Morphological analysis was performed using transmission electron microscopy (TEM; JEM-2100 F, JEOL Ltd., Japan). The structural characteristics were assessed through Fourier transform infrared (FTIR) spectral data in the 500–4000 cm^−1^ range (VERTEX 80v series, Bruker Co., Ltd., Korea). The dispersion and stability of nanofluids were measured with a UV/Vis spectrophotometer (X-ma 3000 Series Spectrophotometer, Human Co., Ltd., Korea) operating at wavelengths ranging from 250 to 900 nm and a zeta potential analyzer (Malvern Zetamaster, Malvern Instrument Ltd., UK). The thermal conductivity of the nanofluids was assessed using the hot wire transient method with a thermal conductivity analyzer (LAMBDA, F5 Technologies GmbH, Willingshausen, Germany) operating at temperature ranges from 20 to 40 °C, with an interval of 5 °C. The electrical conductivity of nanofluids was measured using a conductivity meter (Model CM-25R, DKK-TOA Co., Ltd., Japan).

### 2.3. Functionalization of MWCNTs

The functionalization of MWCNTs was performed using two different oxidation methods, namely acid treatment and alkaline treatment.

For acid treatment, the surfaces of MWCNTs were modified with a 1:3 volume ratio of concentrated nitric acid (HNO_3_) and sulfuric acid (H_2_SO_4_). Then, 1 g raw MWCNTs was suspended in a 40 mL mixture of acids via ultrasonication (1510E-DTH, Branson Ultrasonic Corporation 41, Danbury, CT 06813, USA) for 40 min at room temperature. Next, the oxidation reaction was performed at 100 °C for 100 min on a magnetic stirrer (hot plate stirrer, SMSH-20A, Scilab Korea., Ltd.). The acidic mixture of MWCNTs containing carboxyl radicals was diluted by distilled water. The sample was filtered and washed until the pH value reached 7.0, then it was dried in a furnace [34]. The acid-treated sample was designed as A-CNT.

**Figure 1:**
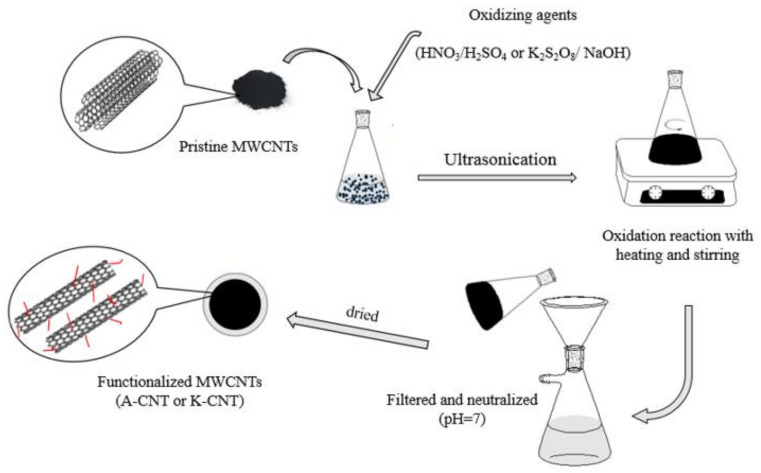
Schematic of the treatment processes.

For alkaline treatment, surface modification of MWCNTs was performed with potassium persulfate (K_2_S_2_O_8_). Then, 1 g pristine MWCNTs and 40 mL distilled water were added to a flask and dispersed with ultrasonic bath for 40 min. Next, 4g K_2_S_2_O_8_ was added to the flask and the pH of the reaction system was adjusted to 13 by adding a concentrated NaOH solution. The flask equipped with a reflux condenser and a magnetic stirrer was kept at 85 °C with mixing for 2 h and then cooled down to room temperature. The contents of the flask were separated by a membrane filter and rinsed with distilled water until a pH of 7 was reached. Lastly, the functionalized MWCNTs were dried in the furnace [33]. The alkaline-treated sample was designed as K-CNT. Figure 1 shows a schematic of the treatment processes. The treated MWCNTs were used for next step of the experiment.

### 2.4. Milling of MWCNTs

A ball mill is one of the most useful methods for the mixing and grinding of raw materials in both laboratory and industry settings [35,36]. A planetary ball mill (HPM-700, Haji Engineering, Korea) was used to grind and shorten the CNTs. There were some factors we needed to be considered. First, the size of the grinding ball. Smaller balls increase the number of contact points between balls during the grinding process. However, the grinding performance decreases due to a reduction of kinetic collision energy [37]. Then, regarding the speed of rotation, higher speeds make the balls move strongly and violently, leading to greater improvements of the grinding efficiency [38]. Finally, the grinding time must be considered, since it is known that the size of the particles decreases with an increase in time [39]. Munkhbayar et al. [40] compared the dry and wet grinding conditions at various rotation speeds (200–500 RPM) of a planetary ball mill regarding the dispersion characteristics of MWCNTs in aqueous solution. It was found that the ground particles under the wet conditions and high rotation speed showed good dispersion in the suspension. Based on the previously published literature [40], mono-sized (3.0 mm) spherical zirconia (ZrO2) balls were used as the collision medium in this study, the milling process performed under wet conditions for 1 h, and the agitator-applied rotation speed was 500 RPM. The details of the grinding process were previously described elsewhere [40].

### 2.5. Preparation of MWCNT Nanofluids

The milling process was performed under wet conditions at a rotation speed of 500 RPM for 1 h on raw and previously treated MWCNT structures (CNT, A-CNT, and K-CNT). Then, MWCNT nanoparticles were dispersed in distilled water without any surfactant by ultrasonication (Branson Ultrasonication Corporation 41, Danbury, CT 06813, USA) for 40 min [15]. All of the nanofluids were prepared at a concentration of 0.1 wt%. The dispersion characteristics of MWCNT nanofluids were analyzed using a UV-Vis spectrometer and zeta potential analyzer. The lambda system measured the thermal conductivity of the nanofluid.

## 3. Result and Discussion

### 3.1. Morphological Surface Analysis of MWCNTs

**Figure 2:**
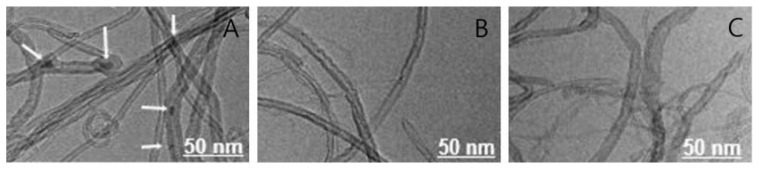
TEM images of the raw and functionalized MWCNTs: (**A**) raw CNT; (**B**) A-CNT; (**C**) K-CNT.

The morphological characteristics of MWCNTs were observed by TEM in this study. Figure 2 shows the TEM images of raw MWCNTs and functionalized MWCNTs under two different conditions. Some particle impurities remained on the carbon nanotube surfaces due to the synthesis process (white arrows in Figure 2A) [41]. In this study, the mixture of concentrated acids and potassium persulfate as the oxidant was used for the treatment processes. Therefore, the structure impurities were removed from MWCNTs and the tips of the nanotubes opened after the chemical processes, which can be clearly seen in Figure 2B,C.

**Figure 3:**
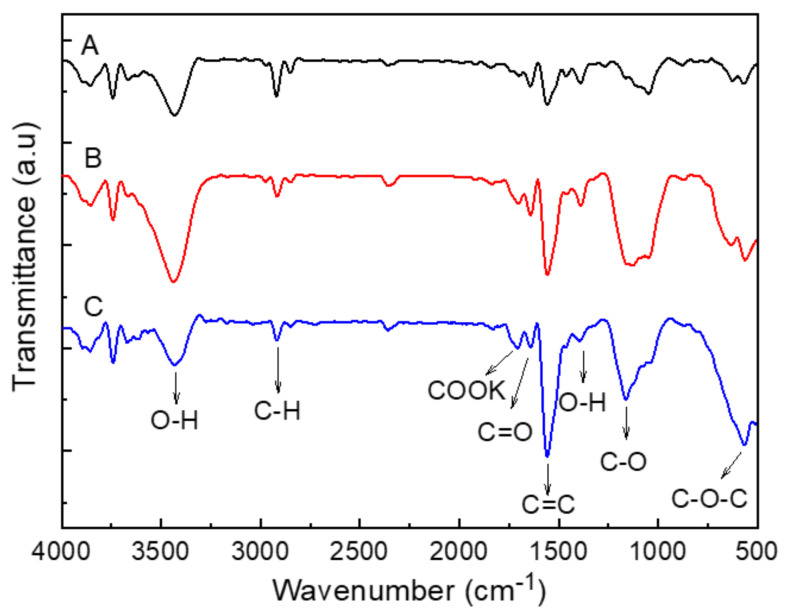
FTIR spectra of pristine and functionalized MWCNTs: (**A**) raw CNT; (**B**) A-CNT; (**C**) K-CNT.

The structural study and the confirmation of the functional groups were carried out using FTIR spectroscopy. FTIR is mainly performed as a qualitative technique for the evaluation of functional groups. Figure 3 shows the FTIR spectra of raw MWCNTs and functionalized MWCNTs under two different conditions. The trends in the FTIR spectrum in Figure 3 were similar, providing similar oxygen functional groups, however the peak strength was different.

The peaks around 3430 cm^−1^ can be attributed to O–H vibrations in the hydroxyl and carboxyl formed after surface treatment processes [34,42]. The O–H stretching intensity of A-CNT was stronger than K-CNT. Potassium persulfate was used for alkaline treatment, so potassium carboxylate (COOK) functional groups were attached to the surfaces of CNTs. Therefore, the O–H stretching intensity was lightly decreased for K-CNT as compared to A-CNT. The bands around 2800–2900 cm^−1^ were caused by the C–H asymmetric and symmetric stretching vibration derived from a long alkyl chain [43]. The absorption peak around at 1715 cm^−1^ corresponded to the stretching vibration of C=O from the carboxylic groups. A characteristic carbonyl peak was also observed around 1635 cm^−1^ and was assigned to the carbonyl group from quinine or the ring structure [22], which shows the low intensity in all of the MWCNT structures. The appearance of the peak around 1550 cm^−1^ showed the existence of carbon double bonding (C=C), revealing the structure of pristine MWCNTs [33], while the peak intensity was increased in all treated MWCNT structures. [44]. We observed that impurities such as amorphous carbon were completely removed from the surfaces of MWCNTs after the treatment processes, and for that reason peak the intensity was increased. The peaks around 1100 cm^−1^ corresponded to the C–O stretching mode of the carboxylic acid group, which had a strong intensity in each modified structure of the MWCNTs [22]. The peaks around 600 cm^−1^ can be assigned to stretching vibration of C–O–C groups [34]. These peaks indicated the successful generation of oxygen functional groups on the nanotubes [34,41].

### 3.2. Dispersion Characteristics of MWCNTs

**Figure 4:**
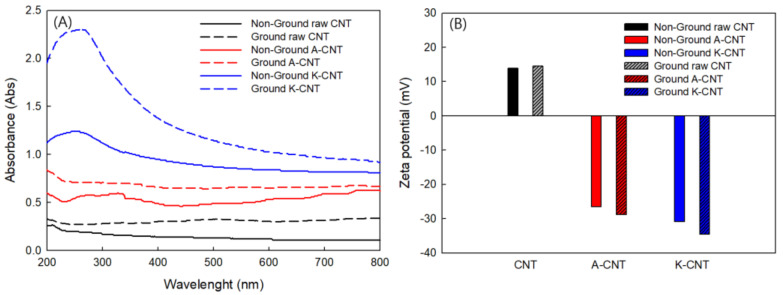
UV-Vis spectra (**A**) and zeta potential (**B**) comparison of non-ground and ground MWCNT nanofluids for raw and functionalized MWCNTs.

Uniform dispersion and stable suspension of nanoparticles in the dispersing media are essential for the applications and final properties of the nanofluids [45,46]. The dispersion of carbon nanotubes can be characterized using UV-Vis spectroscopy [45,46,47,48,49]. Figure 4A shows UV-Vis spectra of suspended pristine and different chemically treated MWCNTs with and without mechanical milling. Two different chemical treatments were employed in the surface modification process, which significantly improved the dispersion of MWCNTs in this study. Chemically treated MWCNTs had significantly improved dispersion when compared to the raw MWCNT suspension. The introduction of oxygen functional groups at nanotube defect sites increases the hydrophilicity of the carbon nanotubes [50,51,52,53], Hydrophilic materials are more efficiently dispersed in aqueous solution; hence, the dispersion of CNTs was greatly improved. Additionally, the van der Waals forces between nanotubes decreased due to the attachment of functional groups, facilitating the separation of nanotube bundles into individual nanotubes [54]. This indicated that the functionalization is essential for the dispersion of MWCNT nanofluids. Further, utilization of the milling process provides improved dispersion of the MWCNT nanofluids. Our previously published studied [40] investigated the planetary ball milling effects on the dispersibility and thermal conductivity of CNTs in aqueous solution. As a result, it was found that the length of the CNTs shortened after grinding, as proven by particle size analysis. The agglomeration propensity was decreased and MWCNTs fibers were shortened by planetary ball milling. Small particles are better dispersed in dispersion media than large particles.

Additionally, the zeta potential is a key indicator of the stability of colloidal dispersions. The magnitude of the zeta potential indicates the degree of electrostatic repulsion between similarly charged adjacent particles in the suspension, so suspensions with high zeta potentials (negative or positive) indicate electrically stabilized particles, while colloids with low zeta potentials tend to coagulate or agglomerate [55]. The zeta potentials of pristine MWCNTs and surface-modified MWCNT suspensions with milling effects are shown in Figure 4B. After oxidative treatment of the pristine MWCNTs, the zeta potential of the nanofluid changed from positive to negative due to the formation of negatively charged oxygen functional groups on the surfaces of the MWCNTs. The magnitude of the zeta potential of functionalized MWCNT nanofluid greatly increased compared to pristine MWCNT nanofluid. Electrostatic repulsion between the nanotubes increased due to the formation of functional groups on the surfaces of MWCNTs; therefore, agglomeration of MWCNTs was reduced, however the stability of the MWCNT nanofluid was maintained. In addition, the zeta potential of milled and pristine MWCNTs was compared in aqueous solution (Figure 4B). The zeta potential of milled MWCNT nanofluid was slightly higher compared to the raw MWCNT nanofluids. The increase of the zeta potential was due to the lighter weight of the MWCNTs particles, the shortened lengths, and the reduced agglomeration that resulted from the milling process. In this study, the zeta potential of the raw MWCNT suspension was 13.87 mV (positive). After functionalization and milling processes, the zeta potentials increased to 28.8 mV and 34.6 mV (negative) for milled A-CNT and K-CNT nanofluids, respectively. When the particle size is small enough, high electrostatic repulsion will confer stability. In suspensions where the zeta potential is close to zero (isoelectric point), particles tend to agglomerate. At highly negative or positive zeta potential values (more than 30 mV or less than −30 mV), particles in the suspension tend to repel each other and no agglomeration occurs [56].

**Figure 5:**
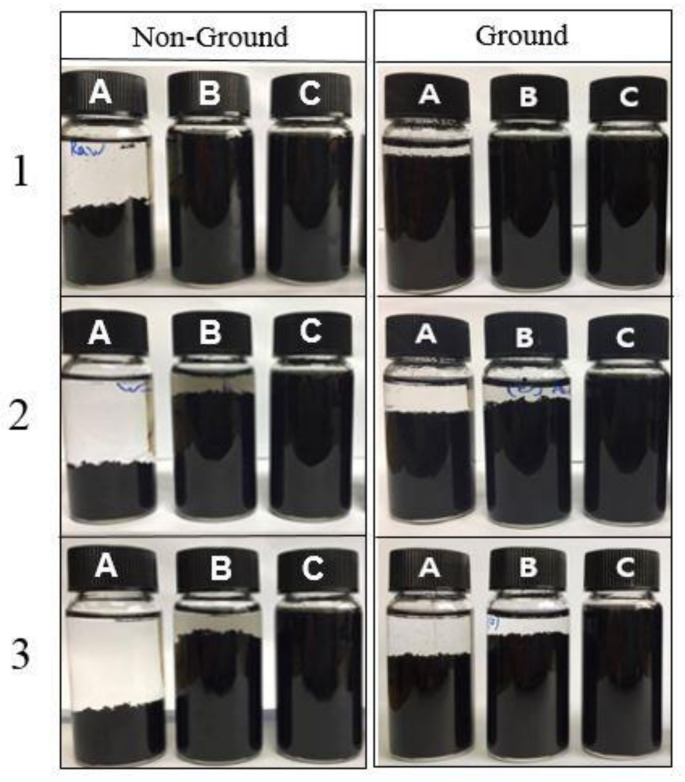
Photographs of pristine and functionalized MWCNT nanofluids for non-ground and ground MWCNTs: (A) raw CNT; (B) A-CNT; (C) K-CNT; (**1**) after sonication; (**2**) after 7 days; (**3**) after 30 days.

Figure 5 shows photographs of the samples at different timepoints. The raw MWCNTs agglomerated rapidly after sonication—agglomeration of A-CNT started after 1 day. The K-CNT nanofluid was stable with no visible aggregation even after one month. From the UV-Vis spectra, zeta potential, and visible tests, the dispersion characteristics of K-CNT were higher than A-CNT. For K-CNT, the K_2_S_2_O_8_ oxidant generated potassium carboxyl groups (COOK) on the exterior of the nanotubes, whereas carboxyl groups (COOH) were generated on nanotubes after acid treatment. Zhang et al. [57] revealed that the COOH on nanotube surfaces is not easily ionized in aqueous solution as compared to COOK; therefore, COOK functional groups are strongly ionized. The hydrophilic nanotubes most probably resulted from the formation of COOK under alkaline treatment. The greatest dispersion characteristics were obtained for milled K-CNT. Therefore, the combination of alkaline treatment and the milling method were more effective for improving dispersion.

### 3.3. Thermal Conductivity of MWCNTs

**Figure 6:**
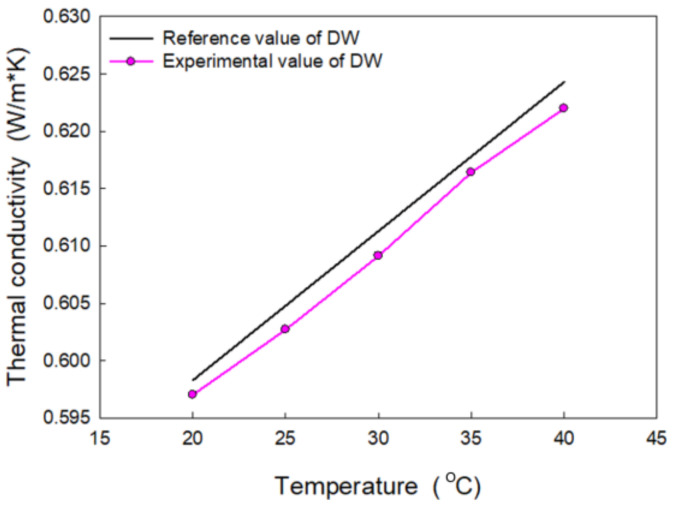
Thermal conductivity measurements of distilled water: experimental data and reference data.

The thermal conductivity of the fluid is an important transport property in terms of the practical applications. The thermal conductivity of nanofluids with respect to temperature is presented in this section. The LAMBDA system was calibrated with DW before the measurement. The calibration result was compared with the reference data presented in the standard textbook [58]. As shown in Figure 6, the result shows relatively good concurrence between the measurement and reference values—the measurement uncertainty was within 0.4%.

**Figure 7:**
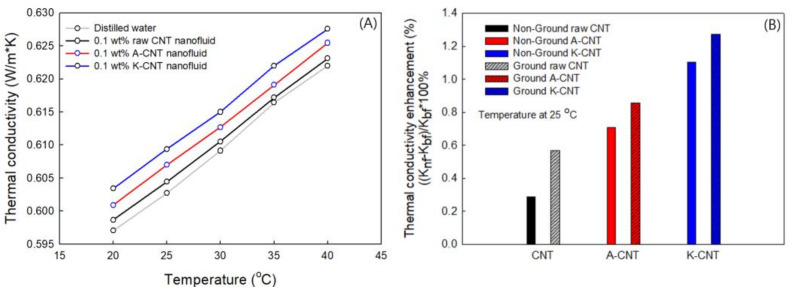
Thermal conductivity of non-ground (**A**), ground and non-ground (**B**) MWCNT nanofluids for pristine and functionalized MWCNTs.

The thermal conductivity was determined as a function of temperature for each MWCNT nanofluid (Figure 7A). The thermal conductivity increased with increasing temperature. As the temperature increased, the viscosity of the nanofluid decreased because of an increase in the Brownian motion of the nanoparticles [59]. It was shown that the thermal conductivity increased due to convection, such as effects the induced by Brownian motion.

The thermal conductivity of the functionalized MWCNT suspension resulting from two different chemical treatments was increased as compared to the raw MWCNT suspension, as shown in Figure 7A. The thermal conductivity of the raw MWCNT nanofluids at a concentration 0.1 wt% were enhanced from 603.5 to 606.9 and 609.4 mW/m·K at 25 °C for A-CNT and K-CNT, respectively. It should be noted that the chemical functionalization significantly improved the dispersion and stability of MWCNTs, thereby increasing the thermal conductivity [60].

Figure 7B shows a comparison of the thermal conductivity levels of pristine and milled MWCNT nanofluids at 25 °C. After milling processes, the thermal conductivity was slightly increased in each MWCNT nanofluid. The improvement of thermal conductivity can be explained by the increase of the specific surface area of the suspended nanostructure due to a decrease of the size of the agglomerated particles by the milling process. The shape and dimensions of nanoparticles can be affected by the thermal conductivity of the nanofluid [54]. It has been shown that shortening of the length of nanotubes and decreasing particle size are important steps in terms of the magnitude of thermal conductivity enhancement. The thermal transfer of the short structures can be faster than for long particles [61], meaning that energy transport inside the liquid is strong and the thermal conductivity increases. The greatest thermal conductivity enhancement obtained for the ground K-CNT was 1.3% (0.610 W/m·K) compared to base fluid (water 0.602 W/m·K).

### 3.4. Electrical Conductivity of MWCNTs

The electrical conductivity is a unique property for electromechanical devices and microelectronics. To confirm the dispersion characteristics, an electrical conductivity experiment was conducted in this study. Each MWCNT structure was analyzed. Before the electrical conductivity measurement, the calibration was applied with a potassium chloride standard (1.41, 12.86 μS/cm) solution.

**Figure 8:**
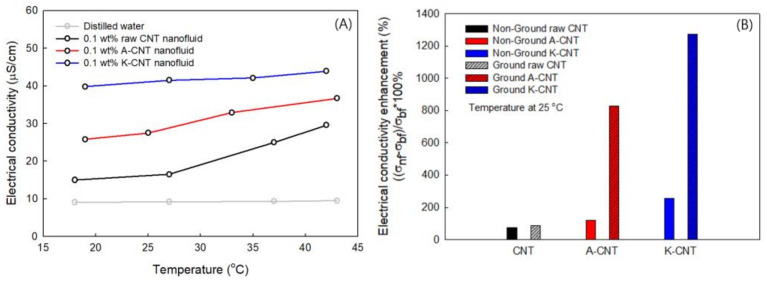
Electrical conductivity of non-ground (**A**), ground and non-ground (**B**) MWCNT nanofluids for pristine and functionalized MWCNTs.

Figure 8A shows the electrical conductivity of the nanofluids at 0.1 wt% concentration as a function of temperature. The electrical conductivity increased with increasing temperature for the treated MWCNT nanofluids. The electrical conductivity of functionalized MWCNT nanofluids was higher than for the raw MWCNT nanofluid.

An important factor affecting the electrical conductivity is the agglomeration of the nanoparticles in the base fluid [62]. The chemical functionalization significantly reduced the agglomeration of MWCNTs, thereby increasing the electrical conductivity. The electrical conductivity of the MWCNT nanofluid at a concentration of 0.1 wt% was enhanced from 16.19 to 27.4 and 41.2 μS/cm at 25 °C for A-CNT and K-CNT, respectively. The highest electrical conductivity was determined for K-CNT. Therefore, alkaline treatment is more effective for increasing electrical conductivity, meaning that it showed the same result as for the thermal conductivity.

Figure 8B shows a comparison of the electrical conductivity enhancement of non-milled and milled MWCNT nanofluids at 25 °C. After the milling processes, the electrical conductivity was significantly increased for each treated MWCNT nanofluid. The percentage enhancement in electrical conductivity was calculated using the formula [(σ_1_ −σ_0_)/σ] × 100%, where “σ_0_” corresponds to the electrical conductivity of the base fluid and “σ_1_” corresponds to the electrical conductivity of the nanofluid. The highest enhancement was 1275% for the milled K-CNT, with a mass fraction of 0.1% at 25 °C. It can be deduced that the improvement of the dispersion characteristics and the increase in the electrical conductivity are attributable to the increase of the surface area and electrophoretic mobility of the particles [63,64,65].

In this work, it was successfully found that alkaline functionalization and milling processes can effectively promote reduced agglomeration, increased dispersion, and long-term maintenance of the stability of MWCNT nanofluids, thereby improving the properties of MWCNT nanofluids.

Finally, details of the result are shown in Table 1, corresponding to the experimental conditions.

## 4. Conclusions

In this experimental study, surface modifications under different conditions (acid and alkaline treatment) and milling processed were investigated regarding their effects on the dispersion characteristics of the thermal and electrical conductivity of the MWCNTs in aqueous solution.

From the obtained results, the functionalized and ground MWCNTs showed higher dispersion characteristics as compared to the raw MWCNT nanofluid. The dispersion characteristics of the nanofluids were increased by treatment and ball milling processes. The greatest thermal conductivity enhancement for MWCNTs was 1.3% (0.610 W/m·K) as compared to the base fluid (distilled water 0.602 W/m·K) for the ground K-CNT at 25 °C. The greatest electrical conductivity enhancement for MWCNTs was 1275% (125.8 μS/cm) compared to the base fluid (9.15 μS/cm) for the ground K-CNT at 25 °C. The results indicated that the properties of alkaline-treated MWCNTs were better than acid-treated MWCNTs. Therefore, the alkaline method is more effective, environmentally friendly, and convenient for the preparation of CNT nanofluids and does not require the use of organic solvents or strong acids. Functionalization alone was not sufficient to improve the dispersion of CNTs in the base fluid. Use of the functionalization and ball milling methods together was necessary to improve dispersion and thermal and electrical conductivity of the MWCNT nanofluids.

## Figures and Tables

**Table 1:** Characteristics of the pristine and surface-modified MWCNTs.

No.	Sample	Type of Agent	Conc. (wt%)	Zeta Pot. (mV)	Thermal Cond. (W/m·K) at 25 °C	Electrical Сond. (μS/cm) at 25 °C
1	Non-ground Pristine CNT	-	0.1	13.87	0.6035	16.19
2	Ground Pristine CNT	-	0.1	14.54	0.6061	17.32
3	Non-ground A-CNT	H_2_SO_4_/HNO_3_	0.1	−26.6	0.6069	20.2
4	Ground A-CNT	H_2_SO_4_/HNO_3_	0.1	−28.83	0.6078	85.1
5	Non-ground K-CNT	K_2_S_2_O_8_/NaOH	0.1	−30.9	0.6094	32.7
6	Ground K-CNT	K_2_S_2_O_8_/NaOH	0.1	−34.57	0.6104	125.8

## Data Availability

Data is contained within the article.
